# P-1789. Emergence of Salmonella enterica serovar Infantis Carrying the IncFIB (K)_1_Kpn3 (pESI-like) Plasmid in Clinical Isolates in Ecuador

**DOI:** 10.1093/ofid/ofaf695.1958

**Published:** 2026-01-11

**Authors:** Jeannete Zurita, Heydi Tonguino, Gabriela Sevillano, Gabriela Sevillano, Andrés Herrera-Yela, Fernando Lara-Freire, Ariane Paz y Miño, Camilo Zurita-Salinas

**Affiliations:** Unidad de Investigaciones en Biomedicina. Zurita & Zurita Laboratorios, Quito, Pichincha, Ecuador; Biomedical Research Unit. Zurita & Zurita Laboratorios, Quito, Pichincha, Ecuador; Unidad de Investigaciones en Biomedicina. Zurita & Zurita Laboratorios, Quito, Pichincha, Ecuador; Unidad de Investigaciones en Biomedicina. Zurita & Zurita Laboratorios, Quito, Pichincha, Ecuador; Universidad Internacional SEK, Quito, Pichincha, Ecuador; Biomedical Research Unit. Zurita & Zurita Laboratorios, Quito, Pichincha, Ecuador; Mass General Brigham Salem Hospital, Salem, Massachusetts; Unidad de Investigaciones en Biomedicina. Zurita & Zurita Laboratorios, Quito, Pichincha, Ecuador

## Abstract

**Background:**

Whole-genome sequencing (WGS) has revolutionized Public Health Microbiology and has been used routinely for identification, epidemiological surveillance and monitoring antimicrobial resistance (AMR). In this study, in silico gene detection and clonality analysis identified the presence of multidrug-resistant *Salmonella enterica* serovar Infantis isolates with extended-spectrum β-lactamase AMR determinants harbored on a megaplasmid (pESI-like plasmid) called IncFIB (K)_1_Kpn3 in the Ecuadorian population.

**Figure:**
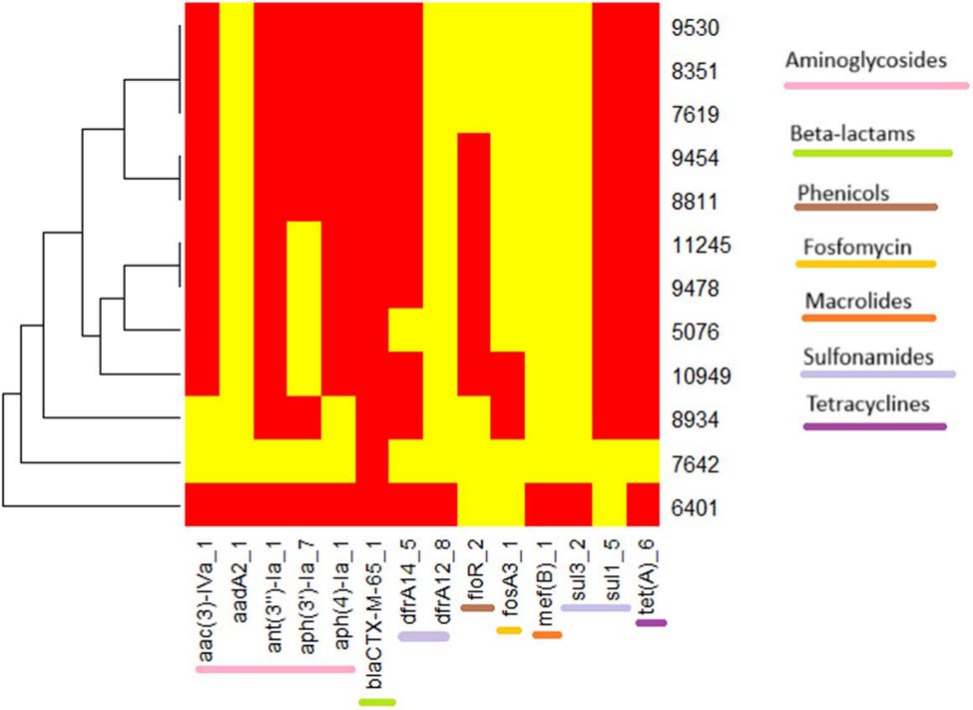
Heatmap of multidrug-resistant strains of Salmonella enterica serovar Infantis. Red = Resistance and yellow = Susceptibility. Plasmid IncFIB (K)_1_Kpn3 diagram in Ecuadorian strains

**Figure:**
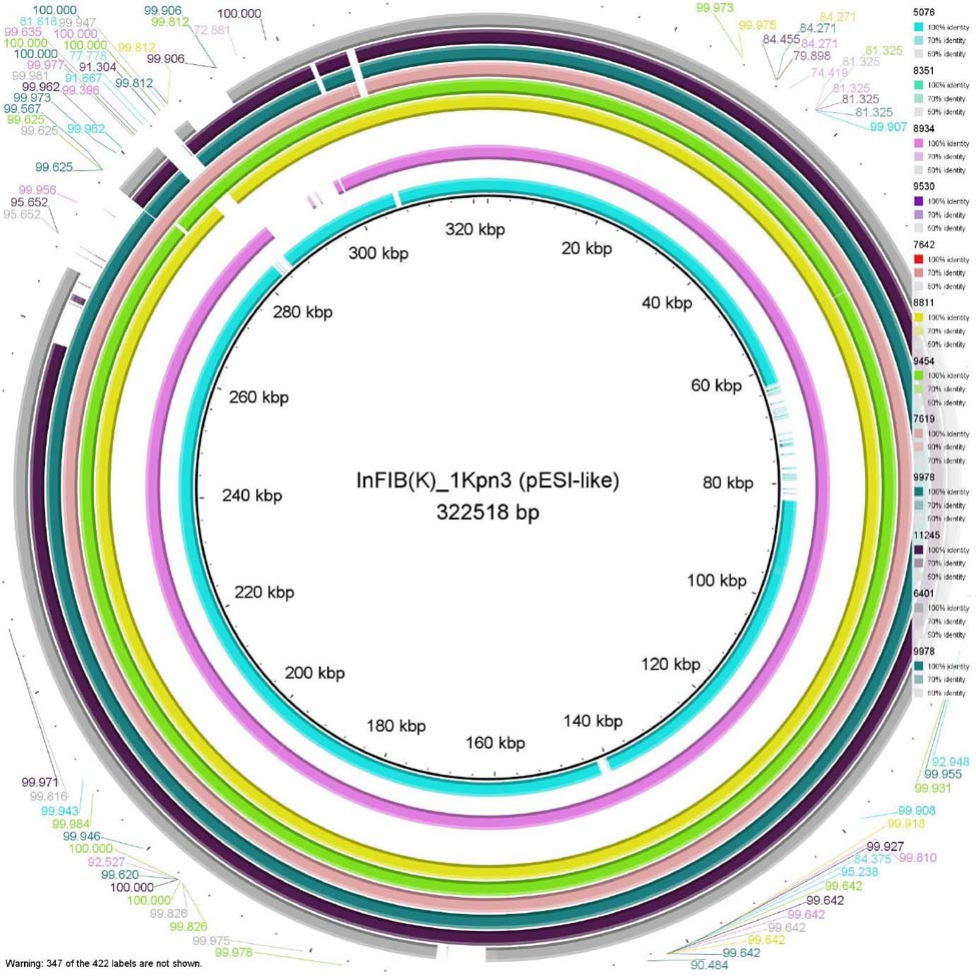

**Methods:**

Twelve S. Infantis strains were obtained from urine cultures of patients aged 9 months to 95 years (11 female, 1 male) between 2022-2024. WGS was performed using Oxford Nanopore MinION (Rapid Barcoding Kit 24 V14 (SQK-RBK114.24). Data were processed using Galaxy Australia: Porechop for trimming, Flye for de novo assembly, and Abricate (Virulence Factor Database, ResFinder, and PlasmidFinder).

**Results:**

The presence of *bla*CTX-M-65, amongst other resistance determinants conferring resistance to β-lactams, macrolides, aminoglycosides, tetracycline, sulfonamide, phenicol and fosfomycin were identified (Figure 1). Analyses of the genome indicated that the resistance were located on a 322518 bp plasmid that showed 99 % sequence similarity to an IncFIB plasmid (CP016409) (Figure 2). Clonality analysis showed that 100% isolates were belong to clone ST32 and all harbored the IncFIB plasmid. Furthermore, this plasmid contained genes associated with virulence, such as *ybt* (iron uptake), *fae* (fimbriae) which enhance the colonization capability and fitness of the bacterium.

**Conclusion:**

The emergence of the multidrug-resistant (MDR) ESBL-producing *S. enterica* serovar Infantis has been linked to the presence of a unique pESI or pESI-like megaplasmid that enhances bacterial adaptation. The presence of pESI-like plasmids has demonstrated the emergence of a stable, resistant ST32 clone that has been circulating in the Ecuadorian population for some time, probably since 2014 (first reports).

**Disclosures:**

All Authors: No reported disclosures

